# Hereditary alpha-tryptasemia demonstrates relative basophil enrichment without signs of cellular hyperreactivity

**DOI:** 10.1016/j.jacig.2026.100699

**Published:** 2026-04-01

**Authors:** Anna-Karin Johnsson, Ionut Atanasoai, Gunnar Nilsson, Theo Gülen

**Affiliations:** a Department of Medicine Solna, Division of Immunology and Respiratory Medicine, Karolinska Institutet, Stockholm, Sweden; e Department of Medicine Huddinge, Clinical Lung and Allergy Research Unit, Karolinska Institutet, Stockholm, Sweden; b Center for Molecular Medicine, Karolinska University Hospital, Stockholm, Sweden; c Clinical Immunology and Transfusion Medicine, Karolinska University Hospital, Stockholm, Sweden; f Department of Respiratory Medicine and Allergy, Karolinska University Hospital, Stockholm, Sweden; d Department of Medical Sciences, Uppsala University, Uppsala, Sweden

**Keywords:** Hereditary alpha-tryptasemia, tryptase, basophils, basophil function, mast cells, FcεRI, MRGPRX2, fMLP, anaphylaxis, systemic mastocytosis

## Abstract

**Background:**

Hereditary alpha-tryptasemia (HαT) is an autosomal dominant trait caused by increased tryptase alpha/beta 1 (*TPSAB1*) copy number, resulting in elevated serum tryptase levels. Although often asymptomatic, HαT is associated with anaphylaxis, flushing, and connective tissue abnormalities. Although mast cells are primarily implicated, basophil involvement in HαT remains poorly defined.

**Objective:**

Our aim was to compare basophil proportions, MRGPRX2 expression, and responsiveness to IgE-dependent and IgE-independent activation in individuals with HαT, individuals with indolent systemic mastocytosis (ISM), and healthy controls (HCs).

**Methods:**

Peripheral blood was obtained from individuals with HαT (n = 20), individuals with ISM (n = 31), and HCs (n = 8). Basophils were identified by flow cytometry; relative basophil frequencies and surface expression of FcεRI and MRGPRX2 were assessed. Basophil activation was evaluated by CD63 upregulation following stimulation with *N*-formylmethionyl-leucyl-phenylalanine, anti-FcεRI antibody, mastoparan, and compound 48/80.

**Results:**

Relative basophil proportions were higher in subjects with HαT than in subjects with ISM. FcεRI surface expression was preserved in those with HαT but reduced in those with ISM, whereas MRGPRX2 expression was not detected at functionally relevant levels. Basophils from individuals with HαT displayed nonresponsiveness to anti-FcεRI more frequently. In contrast, response to formylmethionyl-leucyl-phenylalanine was higher in subjects with ISM than in subjects with HαT and showed a trend of being higher than in HCs. Mastoparan- and compound 48/80–induced activation was undetectable across groups.

**Conclusion:**

HαT features enriched basophil frequency but lacks functional hyperreactivity. An increased rate of nonresponse to FcεRI cross-linking distinguishes HαT from ISM, indicating condition-specific FcεRI signaling dysregulation rather than uniform basophil dysfunction in mast cell–associated disorders.

## Introduction

Hereditary alpha-tryptasemia (HαT) is an autosomal dominant genetic trait caused by increased tryptase alpha/beta 1 (*TPSAB1*) copy number, resulting in elevated baseline serum tryptase levels.^1^ Although many individuals are asymptomatic, some experience symptoms such as anaphylaxis, flushing, or connective tissue abnormalities.^2^ Despite its prevalence (∼5% of populations of European descent in Europe and the United States),^2^^,^^3^ the impact of HαT on effector cell function remains poorly understood.

HαT is considered part of a broader spectrum of mast cell (MC)-associated conditions. These include clonal disorders such as mastocytosis,^4^^,^^5^ among which indolent systemic mastocytosis (ISM) is a well-characterized entity, typically presenting with a low disease burden and a generally favorable prognosis.^6^ Although both HαT and ISM share elevated serum tryptase levels, their underlying mechanisms, namely, gene-dosage effects in HαT versus clonal expansion in ISM, contrast. This raises the question of whether HαT confers altered immune effector cell activity beyond that of MCs.

Basophils, which share functional similarities with MCs, including IgE-mediated activation via FcεRI, responsiveness to bacterial-derived peptides such as *N*-formylmethionyl-leucyl-phenylalanine (fMLP), and expression of tryptase (although at a much lower level than MCs^7^^,^^8^), remain understudied in the context of HαT. Because HaT is associated with more severe anaphylaxis, it is important to understand whether there is a link to *TPSAB1* copy number and basophil reactivity. Basophil counts are generally preserved in mastocytosis, reflecting the disease’s selective clonal expansion of MCs. Although anti-FcεRI–mediated basophil activation appears unaffected (as demonstrated by histamine release assays),^9^ enhanced responsiveness to fMLP has been observed,^10^ indicating a shift in basophil reactivity toward alternative signaling pathways in mastocytosis.

We therefore investigated basophil abundance, receptor expression, and activation responses in individuals with HαT versus in individuals with ISM and healthy controls (HCs). We used flow cytometric analysis to determine whether HαT confers hyperreactivity or altered distribution of effector cells.

## Results and discussion

**Fig 1:**
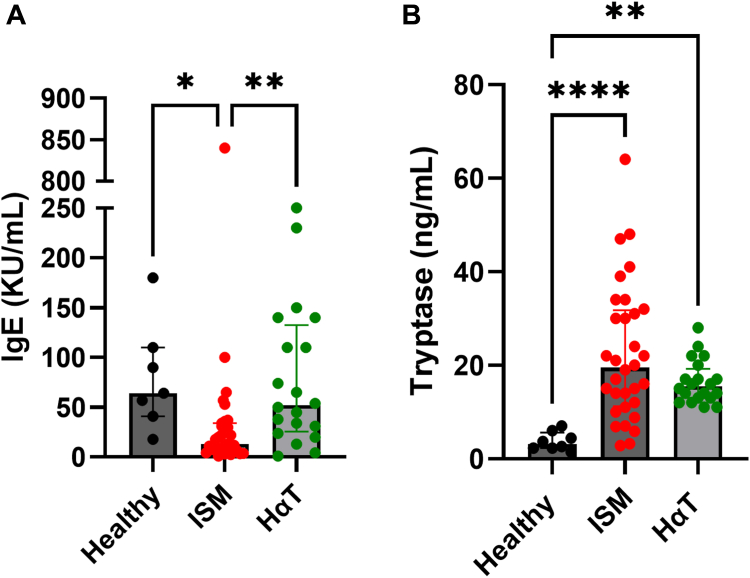
Baseline levels of total serum IgE (**A**) and tryptase levels (**B**) across all groups. Bars in graphs represent medians plus or minus interquartile ranges. ∗*P* < .05; ∗∗*P* < .01; and ∗∗∗*P* < .001. Group differences were assessed using the Kruskal-Wallis test followed by the Dunn *post hoc* test for multiple comparisons in this figure and in all of the following group analyses.

The study subjects were recruited from a clinical cohort with suspected MC-associated conditions. Peripheral blood was collected from 20 individuals with HαT, 31 patients with ISM, and 8 HCs. The Regional Ethics Committees in Stockholm approved the study (ethical approval registration number Dnr. 2009/2082-31/2 and with amendment Dnr. 2018/2618/32), and all participants provided written informed consent. Patient characteristics at the day of sampling are presented in Table E1 (see the Online Repository at www.jaci-global.org). Overall, 52% of patients with ISM and 40% of those with HαT had experienced anaphylaxis (see Table E1). All individuals diagnosed with ISM or HαT were managed with H1 receptor antagonists on an as-needed basis. Baseline levels of total serum IgE and tryptase were measured (Fig 1, *A* and *B*). We compared basophil proportions and functional responses among individuals with HαT and those with ISM, alongside HCs. In the following text, we detail cellular representation, receptor expression, and functional responses and discuss their relevance to HαT pathobiology.

**Fig 2:**
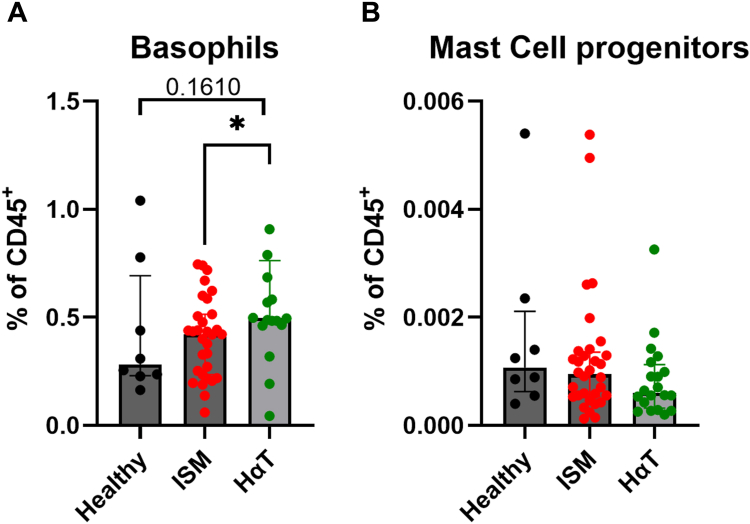
Proportions of basophils (**A**) and MC progenitors (**B**) across all cohorts.

Basophil proportions among leukocytes were significantly higher in patients with HαT than in those with ISM (Fig 2, *A*). Absolute basophil counts were not assessed; therefore, potential differences in total basophil numbers could not be assessed from this study. In contrast, MC progenitor frequencies did not show comparable trends (Fig 2, *B*). This relative enrichment may reflect an altered lineage output, turnover, or survival. Further investigation into the cytokine milieu and bone marrow niche dynamics is needed to determine whether this skew is intrinsic to hematopoiesis or an adaptive response driven by systemic factors.

**Fig 3:**
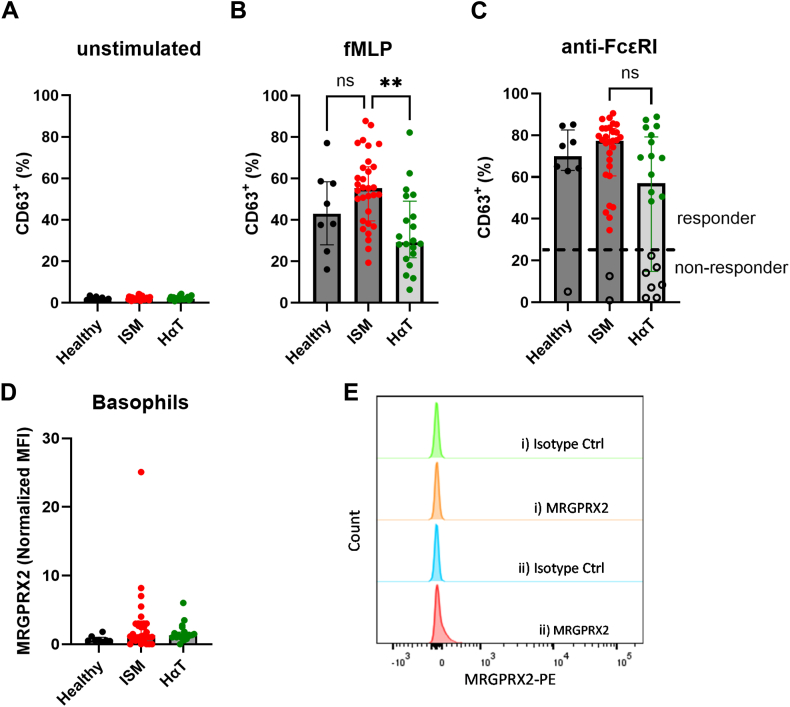
Unstimulated control (**A**) and basophil responsiveness to fMLP (**B**) and anti-FcεRI (**C**) measured as the percentage of basophils displaying surface expression of CD63 (the unstimulated control sample was gated as CD63^–^, and the percentage of CD63^+^ was determined in the stimulated samples by using this gate). **C,** No significant overall group effect was detected, but there is an association between groups and responder versus nonresponder to FcεRI cross-linking (Fisher exact test *P* = .02). In the case of nonresponders, levels of CD63^+^ of 22.3% or lower are denoted by black circles. **D,** Baseline basophil MRGPRX2 surface expression normalized to the isotype control. **E**, Representative expression in a single individual (**i**) and the highest measured expression (**ii**) (in an individual with ISM).

Basophils were identified by flow cytometry, and CD63 expression assessed after treatment with fMLP, mastoparan, compound 48/80, and anti-IgE. The CD63 gate was set by using the basal levels of an unstimulated control (Fig 3, *A*). Additional details on methods are available in the Supplementary Methods in the Online Repository (available at www.jaci-global.org). Basophil responses to fMLP were significantly reduced in patients with HαT versus in patients with ISM, with a similar but not significant trend versus in HCs (Fig 3, *B*). This finding is noteworthy given that patients with ISM are reported to have increased fMLP responsiveness owing to elevated expression of formyl peptide receptor 1 (FPR1).^8^ Notably, nonresponsiveness to FcεRI cross-linking (cutoff 22.3%) was observed in a significantly greater proportion of subjects with HαT (35%) than either in subjects with ISM (6.25%) or in subjects with HCs (12.5%), with a significant association between groups and response-versus-nonresponse to anti-FcεRI (Fisher exact test *P* = .02) (Fig 3, *C*). Taken together, our data establish a divergence: basophils from patients with ISM are quantitatively unaltered but hyperreactive to fMLP, whereas basophils from patients with HαT are enriched yet functionally restrained in response to FcεRI activation.

Functional evaluation of MRGPRX2, a G protein–coupled receptor implicated in non–IgE-mediated MC activation, showed no basophil response to mastoparan or compound 48/80 (data not shown). Absolute surface levels of the receptor were very low and insufficient to drive CD63 upregulation (Fig 3, *D* and *E*). These findings exclude MRGPRX2-mediated mechanisms in HαT and provide the first evaluation of MRGPRX2-mediated basophil activation in patients with HαT or mastocytosis.

**Fig 4:**
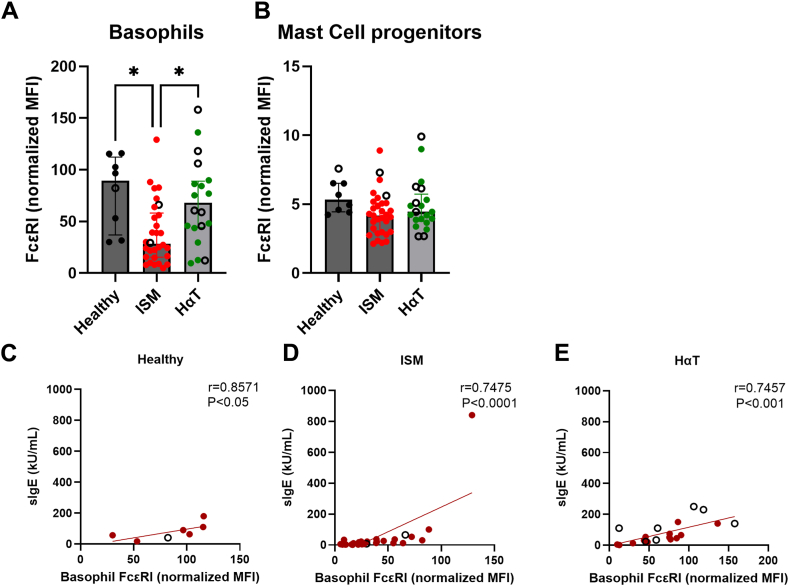
FcεRI surface expression on nonactivated basophils (**A**) and MC progenitors (**B**) and their Spearman correlations to serum total IgE levels, as stratified by group. Healthy individuals with ISM (**D**) and individuals with HαT (**E**).

Next, we evaluated whether the impaired response to FcεRI-mediated activation was explained by receptor loss. Surface FcεRI expression was unchanged in patients with HαT regardless of whether they were responders or nonresponders to FcεRI cross-linking; however, it was partially downregulated in patients with ISM, although without eliciting an altered response (Fig 4, *A*). This suggests that intracellular signaling constraints, rather than receptor availability, underlie the FcεRI phenotype in HαT. FcεRI surface expression was not statistically different on MC progenitors (Fig 4, *B*). Moreover, total serum IgE levels correlated with FcεRI surface expression on basophils across all 3 groups (Fig 4, *C-E*).

Collectively, individuals with HαT exhibited a higher frequency of nonresponsiveness to FcεRI-mediated stimulation, whereas patients with ISM showed increased reactivity to fMLP that was statistically significant only in comparison with that among those patients in the group with HαT. Although overall activity in patients with HαT aligned with the physiologic range observed in healthy individuals, the frequent occurrence of nonresponders to FcεRI cross-linking, which was observed in 35% of subjects with HαT versus in approximately 10% of HCs, offers a new perspective on HαT pathobiology. Previous studies have linked such nonresponsiveness to impaired spleen tyrosin kinase (Syk)-dependent signaling,^11^ suggesting an anergic or tolerized phenotype in patients with HαT.

Although our investigation focused on basophils, interpretation of the immunologic profile of patients with HαT requires attention to MCs, given their prominent role in symptomatology and serum tryptase level elevation. Despite reports of clinical symptoms attributed to MC mediators, such as flushing, gastrointestinal discomfort, and anaphylaxis, current understanding suggests that elevated serum tryptase levels in patients with HαT reflect increased *TPSAB1* gene copy number rather than MC activation. This notion is supported by the findings of a study showing normal urinary MC mediator levels despite patients’ characteristically high basal serum tryptase levels.^12^ Additionally, Chollet and Akin demonstrated that individuals with HαT, including those with mastocytosis, did not consistently differ from HCs in terms of urinary MC activation markers or mediator-related symptoms.^13^ Lyons emphasized that HαT may act as a disease-modifying trait, thus amplifying symptom burden without conferring MC hyperresponsiveness.^14^ Furthermore, Gülen and Akin have discussed the overrepresentation of HαT in mastocytosis and anaphylaxis, underscoring the lack of mechanistic evidence for increased MC activation.^15^ Together, the data indicate that HαT appears to modulate disease expression through quantitative effects rather than direct effector cell activation.

Taken together, the data indicate that basophil frequency is elevated in HαT, without signs of hyperactivity. Instead, HαT has been characterized by a high prevalence of nonresponsiveness to FcεRI-mediated activation, distinguishing patients with HαT from both patients with ISM and HCs. In contrast, patients with ISM exhibited a basophil profile marked by increased reactivity to fMLP and a previously undescribed reduction in FcεRI surface expression on nonactivated basophils.

This preliminary study has limitations. The relatively small number of HCs reduced statistical power for comparisons involving this group. In addition, absolute basophil counts were not measured; future studies incorporating absolute counts will be important to confirm our findings.

In conclusion, our findings support the idea that unlike ISM, HαT reflects a genetically driven alteration in MC-basophil biology, in which elevated tryptase levels result from *TPSAB1* copy number variation rather than from clonal expansion or inflammatory priming. The immune profile of HαT—marked by quantitative changes without qualitative escalation—may reflect lineage bias, epigenetic tuning, or gene dosage effects. Further studies, including longitudinal and single-cell analyses, are needed to clarify the underlying mechanisms.

**Key messages**

• Basophil enrichment is indicated in patients with HαT relative to in patients with ISM.
• A decreased response rate to FcεRI cross-linking in HαT basophils contrasts with the hyperreactive phenotype to fMLP seen in patients with ISM.
• This divergence may guide diagnosis and clarify immune regulation in MC-associated disorders.

## Disclosure statement

Supported grants from the Konsul THC Bergh Foundation, Sweden; the Asthma and Allergy Association’s Research Fund in Sweden; the Swedish Research Council–Medicine and Health, the Swedish Cancer Society; the Ellen, Walter and Lennart Hesselman Foundation; and the Stockholm County Council Research Funds (ALF).

Disclosure of potential conflict of interest: The authors declare that they have no relevant conflicts of interest.

## Supplementary data

Supplementary Table E1
