# Supplementary material for: Hereditary alpha-tryptasemia demonstrates relative basophil enrichment without signs of cellular hyperreactivity
Source: J Allergy Clin Immunol Glob. 2026 Apr 1;5(4):100699. doi: 10.1016/j.jacig.2026.100699 (PMC13123581; doi:10.1016/j.jacig.2026.100699)
Supplement: Supplementary Table E1 [file mmc1.docx]

**Table E1:** Demography and clinical characteristics of study subjects and controls at the day of the study

| Characteristics | ISM (n=31) | HαT (n=20) | HC (n=8) |
| --- | --- | --- | --- |
| Age at study (y), median (range) | 59 (31-77) | 63 (29-81) | 50.5 (27-72) |
| Gender: male, n (%)  Gender (M/F) | 9 (29%)  9/22 | 7 (35%)  7/13 | 4 (50%)  4/4 |
| Baseline tryptase (ng/mL), median (range) | 19 (2.8-64) | 15.5 (11-28) | 3.2 (1.4-7) |
| Total IgE (kE/L), median (range) | 13 (1-840) | 52 (2-250) | 64 (18-180) |
| Subjects with anaphylaxis, n (%) | 16 (52%) | 8 (40%) | 0 |

Abbreviations: ISM, indolent systemic mastocytosis; HαT, hereditary alpha-tryptasemia; HC, healthy controls
