# Supplementary material for: Hereditary alpha-tryptasemia demonstrates relative basophil enrichment without signs of cellular hyperreactivity
Source: J Allergy Clin Immunol Glob. 2026 Apr 1;5(4):100699. doi: 10.1016/j.jacig.2026.100699 (PMC13123581; doi:10.1016/j.jacig.2026.100699)
Supplement: Supplementary Methods [file mmc2.docx]

**Materials and Methods**

**Study Samples**

The study was approved by the regional ethical committee (DNR: 2009/2082-31/2; and amendment DNR: 2018/2618/32). After receiving informed consent peripheral blood was collected from study participants in EDTA-treated tubes, around the same time of the day throughout the study. Blood samples were kept at room temperature and processed within 24 h of sampling for flow cytometry-based assays.

**Basophil Activation Test (BAT)**

Basophil activation was assessed by flow cytometry using the Flow CAST kit (FK-CCR, Bühlmann Laboratories) according to the manufacturer’s instructions. Four stimulation conditions were included: fMLP (kit concentration), anti-FcεRI (kit concentration), Mastoparan (25 µM final), and Compound 48/80 (1 µg/mL final) also unstimulated control was included and used for gate setting in flow cytometry analysisWhole blood (50 µL per condition) and staining reagent were added to stimulation tubes (final volume 220 µL per tube). Samples were incubated at 37 °C for 25 minutes, followed by the addition of 2 mL lysing reagent, gentle mixing, and incubation for 10 minutes at room temperature. Cells were pelleted at 500 × g for 5 minutes and resuspended in 400 µL fixation buffer (BioLegend). After 15 minutes at 4 °C, samples were washed twice with 2 mL cold PBS, centrifuged, and resuspended in wash buffer for flow cytometry acquisition.

**Basophils, Mast Cell Precursors (MCp), and MRGPRX2 Expression**

Sample Preparation. EDTA-treated blood was transferred to 15 mL Falcon tubes and the red blood cells lysed with 1.5× volume of BD Pharmlyse buffer (diluted to 1× from stock). Samples were vortexed, incubated for 5 minutes at room temperature, and centrifuged at 259 × g for 3 minutes. Supernatants were discarded, and 5 mL of lysing buffer was added, vortexed, and centrifuged again. Cells were resuspended in PBS, centrifuged, and finally resuspended in FACS buffer (2% heat-inactivated FCS in PBS with 2 mM EDTA). Cells were counted using trypan blue exclusion. Antibody Staining. Cells (maximum 15×10⁶ per analysis) were pelleted, supernatants removed. Fifty microliters of Brilliant Stain Buffer (BD 566349) were added to each tube. The following antibody panel was used (5 µL per antibody per test) linage markers [[CD14-BV510 (M5E2), CD3-BV510 (HIT3a), CD19-BV510 (HIB19)], CD117-APC, CD45-APC-H7, CCR3-FITC, MRGPRX2-PE (K125H4) or PE Mouse IgG2b, κ Isotype Ctrl, CD34-PECy7, FcεRI-BV421. Compensation beads were stained in parallel. Cells were incubated for 30 minutes at 4°C in the dark, washed twice with FACS buffer (400 × g, 5 minutes), and resuspended in FACS buffer. Just before acquisition, 7-AAD (Thermo Fisher #A1310) was added at 1:1000 dilution. Samples were filtered through a 70 µm strainer before analysis.

**Flow cytometry**

All samples were aquired on a BD FACSCanto II (BD Biosciences). For BAT assays, basophils were from single cells identified as CCR3-PE+/ssc low, ≥200 basophil events were collected per sample. Basophil activation was quantified as the percentage of CD63⁺ cells, with gates set relative to the unstimulated control according to manufacturers instruction with a target CD63+ of <5%, e.g between 2- 2.5% CD63 positive cells, (median in our samples was 2,0 (range 0.9 - 4.4). For the non-activated stained samples around 3×10⁶ total events were collected for the isotype controls and ≥10×10⁶ for samples. Basophils and MCp were gated from live single cells as CD45+/linage-/SSC low/CCR3+/ FcERI+ and CD45+/linage-/CD34+/CD117+/FcERI+ respectively. MRGPRX2+ basophils were identified relative to the matched isotype control. To minimize the panel and avoid sample splitting no isotype control was included for FcεRI, normalized mean fluorescence intensity (MFI) was calculated as a ratio relative to eosinophil FcεRI staining, as eosinophils lack FcεRI expression and are biologically and instrumentally appropriate as negative controls with similar size and background fluorescence as basophils and present in all samples.This analysis was crossvalidated with lymphocytes as negative control, yealding similar result but higher noise. Analysis was performed using FlowJo software (Becton Dickinson).

**Data analysis**

All statistical analyses were performed using GraphPad Prism (San Diego, USA). Group differences were analyzed using the Kruskal–Wallis test, followed by Dunn’s post-hoc correction for multiple comparisons. Non-parametric tests were selected because the data were not normally distributed. Significance thresholds were defined as p < 0.05 (*), p < 0.01 (**), and p<0.001(***). Correlations were evaluated using Spearman’s rank correlation, and associations between categorical variables were assessed with Fisher’s exact test.
